# Quantitative Analysis of Staphylococcal Enterotoxins A and B in Food Matrices Using Ultra High-Performance Liquid Chromatography Tandem Mass Spectrometry (UPLC-MS/MS)

**DOI:** 10.3390/toxins7093637

**Published:** 2015-09-11

**Authors:** Aida Zuberovic Muratovic, Thomas Hagström, Johan Rosén, Kristina Granelli, Karl-Erik Hellenäs

**Affiliations:** Science Department, National Food Agency, Box 622, Uppsala SE-751 26, Sweden; E-Mails: thomas.hagstrom2@gmail.com (T.H.); johan.rosen@slv.se (J.R.); kristina.granelli@slv.se (K.G.); karl-erik.hellenas@slv.se (K.-E.H.)

**Keywords:** staphylococcal enterotoxins, quantification, foods, UPLC-ESI-MS/MS

## Abstract

A method that uses mass spectrometry (MS) for identification and quantification of protein toxins, staphylococcal enterotoxins A and B (SEA and SEB), in milk and shrimp is described. The analysis was performed using a tryptic peptide, from each of the toxins, as the target analyte together with the corresponding ^13^C-labeled synthetic internal standard peptide. The performance of the method was evaluated by analyzing spiked samples in the quantification range 2.5–30 ng/g (*R^2^* = 0.92–0.99). The limit of quantification (LOQ) in milk and the limit of detection (LOD) in shrimp was 2.5 ng/g, for both SEA and SEB toxins. The in-house reproducibility (RSD) was 8%–30% and 5%–41% at different concentrations for milk and shrimp, respectively. The method was compared to the ELISA method, used at the EU-RL (France), for milk samples spiked with SEA at low levels, in the quantification range of 2.5 to 5 ng/g. The comparison showed good coherence for the two methods: 2.9 (MS)/1.8 (ELISA) and 3.6 (MS)/3.8 (ELISA) ng/g. The major advantage of the developed method is that it allows direct confirmation of the molecular identity and quantitative analysis of SEA and SEB at low nanogram levels using a label and antibody free approach. Therefore, this method is an important step in the development of alternatives to the immune-assay tests currently used for staphylococcal enterotoxin analysis.

## 1. Introduction

Bacterial protein toxins, such as enterotoxins from *Staphylococcus aureus* (*S. aureus*), are important in the area of food safety as they induce more or less serious food-related illnesses worldwide [1,2,3,4]. Such protein toxins could also represent a potential risk for human health if they were to be produced for intentional abuse [5,6]. The health effects caused by enterotoxins can be observed already at very low doses, although the symptoms are usually milder in healthy adults and comprise nausea, vomiting and diarrhea, while for small children, elderly people and immunosuppressed persons, the effects can be fatal [7]. As the ingestion of enterotoxins has a potential to incapacitate, they also represent an obvious security risk. More than 20 staphylococcal enterotoxins (SEs) are known today, of which only few have been proven to induce illness [4,7]. The ingested quantities reported to cause illness are as small as 100 ng (0.5 ng/mL) [8]. The development of methods for the analysis of SEs is therefore an important issue. There are several types of approaches for the analysis of SEs in foods [9,10,11], among which immunoaffinity methods based on enzyme immunoassay (EIA) comprising enzyme-linked immunosorbent assay (ELISA) and enzyme-linked fluorescent assay (ELFA) [12,13] as well as reverse passive latex agglutination (RPLA) [14,15] are the most common ones. For such methods, there are commercial kits available for detection and confirmation of multiple SEs at low and subnanogram/gram levels [16,17]. Despite the high sensitivity, the immunoaffinity-based methods prerequisite existence of a specific antibody for detection of each of the enterotoxins, which are, so far, only available for a few of the enterotoxins, staphylococcal enterotoxin A to E, G, H and staphylococcal enterotoxin-like Q (SEA-SEE, SEG, SEH and SElQ) [7,18]. The development of anti-enterotoxin antibodies is associated with difficulties and high costs as well as the production of specific antibodies require highly pure toxins, which are not easy to obtain. Along with the difficulties of producing antibodies, the immunoaffinity-based methods are known to suffer from interferences with molecules that have similar properties as the target analyte which causes cross-reactivity and, consequently, lack of specificity for the analysis. This problem is increasingly emphasized with increased sample complexity of different food extracts [19,20]. The need for the development of new methods to circumvent the above-described difficulties has thus been recognized recently. For this purpose, chemical methods using liquid chromatography coupled to electrospray tandem mass spectrometry (LC-ESI-MS/MS) can be good alternatives. Several successful reports employing LC-MS/MS for quantitative analysis of staphylococcal enterotoxins have been reported [21,22,23,24,25]. Different sample preparation strategies have been applied in these studies using food matrices of varying complexity to selectively analyze enterotoxins either as intact proteins or indirectly, in proteomics-based manners, by analyzing their proteotypic peptides after a digestion step. The analyses could be aided by employing immunocapture strategy [22] and/or labeling reagent [22,24].

In this report, we present a label and antibody-free alternative method based on a bottom-up proteomics approach for targeted measurement of SEA and SEB in milk and shrimp with UPLC-ESI-MS/MS. This indirect analysis of toxins uses proteotypic amino acid sequences within the toxin after release by tryptic digestion. The use of corresponding ^13^C-labeled internal standard sequences and tandem mass spectrometry provide high specificity in simultaneous identification and aids the quantification of SEA and SEB toxins in highly complex food extracts at low ppb level. As the method can be applied without the need of specific reagents or antibodies, it may be extended to other food matrices and enterotoxins for which antibodies have not been developed.

## 2. Results and Discussion

Cow-milk (3% fat) and shrimp (without shell) were used in this study as model samples since they represent high-risk foods for *S. aureus* growth and enterotoxin production. From an analytical point of view, these two matrices also represent good model samples for the examination of the method performance for the analysis of target proteins in the presence of high concentrations of matrix proteins and other complex biomolecules found in food. For the analysis of specific proteins in complex samples with high protein content, the different types of proteomics-based approaches used in bioanalysis can also be applied in food-related issues [26,27]. The main challenge when using proteomic strategies is to achieve sufficient sample complexity reduction and compound separation before the final detection, in order to prevent suppression effects in the detection process. Thus, being a crucial part for further analysis, the sample preparation is usually more or less extensive, combining different tools for sample cleanup and enrichment in order to concentrate the analyte to a suitable level at which it can be measured. In the present method, a bottom-up proteomics-based approach has been applied to quantitatively measure enterotoxins in foods. Beside traditional sample preparation steps such as extraction and homogenization, precipitation, and centrifugation, the applied strategy combines a two-step on-filter cleanup and a pre-concentration procedure with a protein degradation step using enzymatic digestion that results in characteristic cleavage products for each protein sequence. The main purpose of the ultrafiltration steps is to increase selectivity in the recovery of enterotoxins from the sample. This is achieved by subsequently using two different filters with molecular range cut-offs above and below the molecular weight range of the staphylococcal enterotoxins (20–30 kDa). According to manufacturer’s recommendations, a factor of three above or below the chosen molecular weight range needs to be chosen to ensure confident recovery, depending on if the retention and/or the flow-through fraction aims to be used. Hereby, the molecules that do not fit within the nominal molecular weight limits (NMWL) window for used filters (3 and 100 kDa) are excluded from further analysis, where their tryptic fragments otherwise would be distributed all over the chromatograms inducing suppression effects in the MS measurements. This facilitates further UPLC-MS/MS analysis of proteolytic peptides and results in the increased sensitivity and specificity in protein identification using MS. Table 1 and Table 2 summarize the amino acid sequences and masses of the tryptic peptides of SEA and SEB, their retention times, charge states and *m*/*z* ratios.

**Table 1:** Summary of tryptic peptides of SEA selected and evaluated for their use in the UPLC-ESI-MS/MS analysis. The three peptides indicated by bold and underlined sequences represent the minimum sequence coverage of 10% (of the amino acid sequence in the protein) required and used for toxin identification in matrices. The bold sequences indicate the peptide used for quantification together with the corresponding ^13^C_6_-internal standard peptide. Qu (quantification fragment), Co (Confirmation fragment).

Toxin	Peptide Sequence	Peptide Mass	Charge State	Q1 *m*/*z*	Q2 *m*/*z*	Retention Time (min)
SEA	GLIVFHTSTEPSVNYDLFGA QGQYSNTLLR	3326.7	3+	1109.9	1454.74	9.2
					1307.67	
					1250.65	
					1179.61	
	GFFTDHSWYNDLLVDFDSK	2305.0	3+	769.4	1165.57	10.0
					1051.53	
					936.50	
					823.42	
	YNLYNSDVFDGK	1433.6	2+	717.83	1157.55	6.3
					1044.46	
					881.40	
					767.36	
	NVTVQELDLQAR	1384.7	2+	693.37	1071.58	6.0
					Qu. 972.51	
					Co. 844.45	
					715.41	
	SELQGTALGNLK	1229.7	2+	615.84	1014.59	5.6
					901.51	
					773.45	
					716.43	
	ESHDQFLQHTILFK	1741.9	3+	581.63	999.60	6.8
					886.51	
					758.46	
					621.40	
	VPINLWLDGK	1153.6	2+	577.83	1055.59	7.8
					958.54	
					845.45	
					731.41	
					618.32	
	QNTVPLETVK	1127.6	2+	564.82	886.52	4.8
					785.48	
					686.41	
					589.36	
Internal standard ISA^13^C_6_	NVTVQELDL[^13^C_6_]QAR	1391.2	2+	696.60	1077.58	6.0
					Qu. 978.51	
					Co. 850.45	
					721.41	

**Table 2:** Summary of tryptic peptides of SEB selected and evaluated for their use in the UPLC-ESI-MS/MS analysis. The three peptides indicated by bold and underlined sequences represent the minimum sequence coverage of 10% (of the amino acid sequence in the protein) required and used for toxin identification in matrices. The bold sequences indicate the peptide used for quantification together with the corresponding ^13^C_6_-internal standard peptide. Qu (quantification fragment), Co (Confirmation fragment).

Toxin	Peptide Sequence	Peptide Mass	Charge State	Q1 *m*/*z*	Q2 *m*/*z*	Retention Time (min)
SEB	SIDQFLYFDLIYSIK	1864.0	2+	932.99	1421.77	11.0
					1274.70	
					1161.62	
					998.56	
	LYEFNNSPYETGYIK	1836.9	2+	919.44	1285.61	6.6
					1171.56	
					1057.52	
					970.49	
	VLYDDNHVSAINVK	1585.5	2+	793.91	1211.60	5.2
					1096.57	
					981.55	
					867.50	
	VTAQELDYLTR	1307.7	2+	654.84	1037.53	6.2
					Qu. 909.47	
					Co. 780.43	
					667.34	
	NLLSFDVQTNK	1277.7	2+	639.84	1051.54	6.9
					938.46	
					851.43	
					704.36	
	YLMMYNDNK	1190.5	2+	596.26	1028.45	5.3
					915.37	
					784.33	
					653.29	
	IEVYLTTK	965.5	2+	483.78	853.47	5.5
					724.42	
					625.36	
	LGNYDNVR	949.5	2+	475.74	837.38	3.4
					780.36	
					666.32	
					503.26	
Internal standard ISB^13^C_6_	VTAQELDYL[^13^C_6_]TR	1314.7	2+	658.33	1043.53	6.2
					Qu. 915.47	
					Co. 786.43	
					673.34	

The enzymatic cleavage procedure was performed in-solution, which was preceded by solubilization of sample proteins using urea combined with denaturing agents. The chosen procedure is based on experiences in previous successful reports for bottom-up proteomics [28,29] and partially adapted for quantification of SEB in particular, according to the work thoroughly carried out by Callahan *et al.* [21]. Investigation of variations in the digestion efficiency was thus considered to be beyond the scope of this work and internal standards were added to the samples prior to the digestion that normalize for the deviations that may occur in this step. Having in mind that the enterotoxins are relatively small, globular proteins known to be extremely stable, the in-solution digestion was a reasonable approach [30] that could improve the protein identification by MS. The enzymatic digestion reaction was controlled by the incubation temperature and the pH (60 °C and pH ≥ 8.2, respectively) over 16 h. In order to achieve maximum digestion efficiency, proteins have to be denatured and reduced since their secondary structures hamper the access for the proteases. Here, urea in ammonium bicarbonate was firstly used to destabilize the native protein structure based on non-covalent bonds, where after the disulfide bonds were cleaved using reducing agent followed by an alkylation step of the resulting cysteine thiol-residues prior to the addition of the enzyme.

The potential target peptides for the analysis of SEA and SEB were chosen by comparison and evaluation of the spectra from multiple reaction monitoring (MRM) experiments. Strong y-type ions were on demand and many of the peptides presented in Table 1 and Table 2 could be potential candidates, although few of them were distinguished by the intensities of their product ions in the MRM spectra appearing in the mass spectral region free from major interferences in both milk and shrimp matrices. Based on these observations, NVTVQELDLQAR (mass 1384.7) and VTAQELDYLTR (mass 1307.7) peptides were selected and used in quantitative analysis of SEA and SEB, respectively. For these two peptides internal standard peptides were synthesized so that all six carbons in leucine, at position nine in each peptide sequence, were exchanged with ^13^C-isotope atoms (NVTVQELD-L[^13^C_6_]-QAR and VTAQELDY-L[^13^C_6_]-TR). This provides internal standards that differ in mass by 6 amu and produce y-ion fragments in MRM with masses 6 amu higher, but with identical chemical properties and the chromatographic retention times compared to the target peptides. Although the cost for the synthesis of a specific internal standard for each of the toxins becomes substantially higher, in comparison to using different surrogate standards, the specificity of the internal standards is of crucial importance for quantitative analysis of target amino acid sequences in highly complex protein matrices. This is especially emphasized concerning the suppression effects in the electrospray MS because of which the similarity of the internal standard with the target peptide needs to be pursued to the highest possible extent. Having in mind that staphylococcal enterotoxins share 40%–90% of the amino acid sequence homology [31], the significance of the remaining sequence variability, in combination with the requirements to ionize and fragment the peptides efficiently, limits the possibilities to find a useful target sequence. In addition, when developing multi-methods for the analysis of enterotoxins in complex food matrices using electrospray MS, for which the present method represents a good platform, the isotope-based internal standards become inevitable prerequisites for a successful quantitative analysis. The ideal approach employs isotope-labeled peptides standards produced by enzymatic digestion from full-length isotope-labeled proteins that compensate for all steps in the analytical method, as shown by Dupuis *et al.* [22], which on the other hand increases the costs and the comprehensiveness of the method and was not reconsidered in the present work.

### 2.1. Validation Design

**Figure 1:**
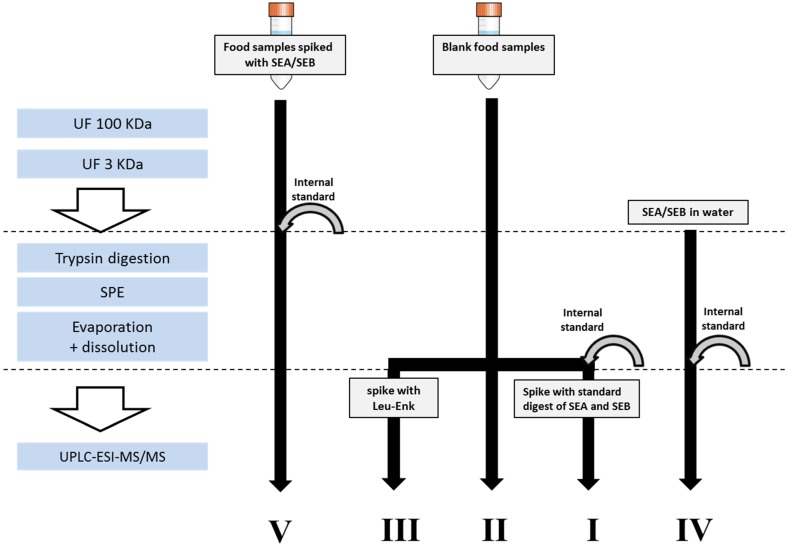
An illustration of the experimental design to evaluate analyte losses during the sample work-up and in the ionization step for the analysis of SEA and SEB in milk and shrimp. Spikings were done using 4–6 different concentrations of SEA and SEB in the concentration range of 2.5–30 ng/g or using corresponding standard digests. Internal standards were ^13^C-labeled target peptide sequences for SEA and SEB, respectively. (1) The peak area quotients **V/I** were used to calculate the absolute recovery of enterotoxins from all sample preparation steps; (2) quotients I/IV were used to calculate the ion suppression (matrix effects) in the ESI for the enterotoxins and for the internal standards; (3) **II** were used to check the specificity of the method (absence of interfering peaks in 15 different types of blank milk); (4) **III** was used as a UPLC-ESI-MS/MS system performance check sample (SPS) together with the Leu-Enk in Milli-Q water; and (5) **IV** and **V** were also used for evaluation of linearity (peak area ratios for toxins and internal standards plotted *versus* SEA or SEB concentrations) and trueness and reproducibility were calculated from repetitions of **V**. Signal intensities for standard peptides produced by enzymatic protein cleavage, **IV**, were approximated to 100% of the initial protein concentration.

To set-up and validate the UPLC-ESI-MS/MS method for quantitative analysis of SEA and SEB toxins cow milk and shrimp samples were spiked with enterotoxin standards and prepared as described in Section 3.3. For the quantitative analysis of SEA and SEB, a synthetic ^13^C_6_-isotope-labeled internal standard peptide for each of the toxins was used. The validation design is partly illustrated in Figure 1.

#### 2.1.1. Specificity

The specificity of the method was investigated using 15 supposedly blank milk samples from different producers that were analyzed regarding the presence of peaks that could disturb or be misidentified as the peaks of the quantification peptides (NVTVQELDLQAR and VTAQELDYLTR) or the internal standards (NVTVQELD-L[^13^C_6_]-QAR and VTAQELDY-L[^13^C_6_]-TR) of SEA and SEB. No interferences were found at concentration levels ≥LOD in any of these samples. The specificity of the method was not tested for different shrimp matrices. Additional experiments for the shrimp matrix are needed in the future to evaluate selectivity and LOQ.

#### 2.1.2. Calibration and Linearity

Two calibration series for milk and shrimp, respectively, were analyzed on different days for evaluation of linearity. The calibration curves were prepared from the analysis of extracts of samples spiked with SEA and SEB, both at toxin concentrations 2.5, 5, 10, 15 and 30 ng/g, prior to sample work up, and internal standards after ultrafiltration but prior to the trypsin digestion step. The derived calibration curves were linear in the tested concentration range for both matrices. The results of the linear regressions are presented in Table 3. Standard curves for digest of SEA and SEB in water were also analyzed and found to be linear (data not shown).

**Table 3:** Linear regression parameters of calibration curves for SEA and SEB in milk and shrimp on two different days. Calibration curves were constructed by plotting the peak area ratios of quantification peptide and internal standard, Area × (IS conc./IS area), *versus* concentration of toxin. Calibration range: 2.5–30 ng/g.

Enterotoxin	Matrix	Calibration Curve		
		Slope	*y*-intercept	*R^2^*
SEA	Milk	0.11	−0.06	0.9582
	Milk	0.10	0.01	0.9858
	Shrimps	0.12	1	0.9563
	Shrimps	0.11	0.62	0.9503
SEB	Milk	0.11	−0.08	0.9183
	Milk	0.11	−0.06	0.9709
	Shrimps	0.19	0.46	0.9645
	Shrimps	0.13	0.06	0.9429

#### 2.1.3. Recovery in Sample Preparation and Matrix Effects in ESI-MS

The important goal in each MS-based protein analysis is an appropriate sample preparation in order to transfer proteins of interest as efficiently and selectively as possible from the sample into a solution for further experimental work in separation and ESI-MS analysis. For the analysis of trace protein concentrations in complex matrices such as foods, this represents a tremendous challenge. This is mainly due to the difficulties to selectively recover the specific analyte proteins in each step of the sample preparation workflow without major analyte losses. A further difficulty is the competitive suppression effects from matrix components in electrospray ionization that can be reduced by proper sample preparation strategy and separation technique. The present approach combines enterotoxin extraction using pH-controlled precipitation to remove excess of proteins, like caseins, followed by filter fractionation of the extract where the components of the highest and lowest molecular weights (Mw) are removed. As the selection of the filter with desired separation properties is essential, the choice of the Mw range was based on the fact that manufacturers determine the Mw cutoffs of the filter membranes with folded rather than denatured proteins. Thus, having in mind that the staphylococcal enterotoxins are globular proteins, which presumably facilitates their passage through the filter membrane, a confident Mw range was preferred to minimize the losses of enterotoxins. Furthermore, the filter fractionation allows for a substantial reduction of the sample extract volume, which makes it possible to combine the part of the sample preparation procedure approved by the EU [32] with the novel strategy. The developed sample preparation procedure is aimed as a general approach to be applicable for extraction of enterotoxins from all types of matrices, which is, in this study, demonstrated using different matrices as milk and shrimp.

In order to estimate the effectiveness of, primarily, the sample preparation procedure, the absolute recoveries of SEA and SEB were calculated from comparison of standard curves in matrix spiked with intact toxins prior to sample preparation and spiked with toxin digest after the sample preparation steps, respectively. Response ratios were measured using area of the quantification peptide fragments (*m*/*z* 972.51 and 909.47) of SEA and SEB or the internal standards fragments (*m*/*z* 978.51 and 915.47). Another experiment was designed to evaluate the matrix effects, *i.e.*, ion suppression, by comparing standard curves in blank matrix spiked with toxin digests after sample preparation and toxin digests in water, respectively. The values of the recoveries and the matrix effects are presented in Table 4 and a depiction of how they were obtained can be found in Figure 1. The recovery values include all the steps in the sample preparation procedure where the contribution of one step to the low recovery, like filter fractionation, might be more significant than the others. The filters used in this work were chosen due to their suitability for concentration and purification of biological samples. Adsorption of proteins to different surfaces during the sample preparation process is, however, a well-known problem in protein analysis [33]. This is probably the most contributing reason for the low recovery also in this study. Although the absolute recoveries were relatively low, the method offers quantification of both SEA and SEB at low ng/g level. Possibilities to refine and adjust the sample preparation procedure to increase the recovery remain open, and the present procedure comprises a good base for further improvements. Similarly, the values in Table 4, calculated both for the SEs and for IS, coherently reflect the expected high ion suppression in the electrospray due to the matrix. This negative impact of the matrix could, to some extent, be reduced by extending the chromatography and thereby reducing the number of components that co-elute with the target peptides [34], which, on the other hand, would prolong the analysis time.

**Table 4:** Absolute recoveries in sample preparation and matrix effects in ESI-MS for spiked milk and shrimp calculated from peak areas. All figures are averages of two repetitions and two injections. For the illustration of the experimental set-up, see Figure 1.

Milk	**SEA Added (ng/g)**	**Recovery in Sample Preparation (%)**	**Matrix Effect Suppression in ESI-MS (%)**	
		**Calculated for SEs (Va/Ia)**	**Calculated for SEs (Ia/IVa)**	**Calculated for IS (Ib/IVb)**
	2.5	2.9	73	73
	5	4.6	75	75
	10	3.5	75	75
	15	4.6	72	74
	**SEB added (ng/g)**			
	2.5	6.1	58	64
	5	6.3	69	65
	10	7.5	68	75
	15	7.3	65	65
Shrimp	**SEA added (ng/g)**			
	10	6.0	75	77
	15	6.3	75	76
	**SEB added (ng/g)**			
	10	6.3	72	72
	15	6.6	74	73

#### 2.1.4. Trueness, Reproducibility, LOD and LOQ

The trueness and in-house reproducibility of the method were calculated from quantitative analysis, including internal standards, of spiked samples at four different concentrations for milk (2.5, 5, 10 and 15 ng/g) and three concentrations for shrimp (2.5, 10 and 15 ng/g). Due to the comprehensiveness of the validation work, the concentrations were determined by using matrix-matched calibration curves based on three concentration levels (0, 8 and 30 ng/g for milk and 0, 5 and 30 ng/g for shrimp). The results, shown in Table 5, were similar for both food matrices, with relative standard deviations (RSD) ranging from 8% to 41% for all the concentration levels and both of the enterotoxins. The limit of detection (LOD) for SEA and SEB in the method was set to 2.5 ng/g and the LOQ to 5 ng/g in shrimp matrix. For milk samples, the LOD was also the LOQ of the method and was experimentally found to be 2.5 ng/g for both SEA and SEB toxins. At the given concentration levels, the toxins could be detected and/or quantified using the criteria described in Section 3.4. These results were based on a short validation study including only a limited number of replicates and concentration levels. Additional repetitive experiments would need to be performed to confirm and refine the findings. For the shrimp matrix, for instance, both SEA and SEB toxins were possible to detect and integrate at 2.5 ng/g, although additional validation experiments are necessary in order to confirm the limit of quantification (LOQ) for the toxins at this concentration level.

**Table 5:** Results from trueness and reproducibility obtained from three and two repetitive validation experiments for milk and shrimp, respectively. In-house reproducibility is based on the analyses done on different days, different persons and different UPLC-MS/MS systems.

Milk	**SEA Added (ng/g)**	***n***	**SEA Found Mean (ng/g)**	**Trueness (%)**	**In house reproducibility RSD (%)**
	2.5	6	1.9	74	22
	5	6	4.6	93	30
	10	6	9.2	92	21
	15	6	15.4	103	9
	**SEB added (ng/g)**				
	2.5	6	2.2	87	23
	5	6	4.7	93	8
	10	6	10.9	109	9
	15	6	18.0	120	11
Shrimp	**SEA added (ng/g)**				
	2.5	2	7	281	9
	10	4	10.6	106	15
	15	4	17.4	116	5
	**SEB added (ng/g)**				
	2.5	2	3.6	143	41
	10	4	8.5	85	25
	15	4	14.8	99	9

**Figure 2:**
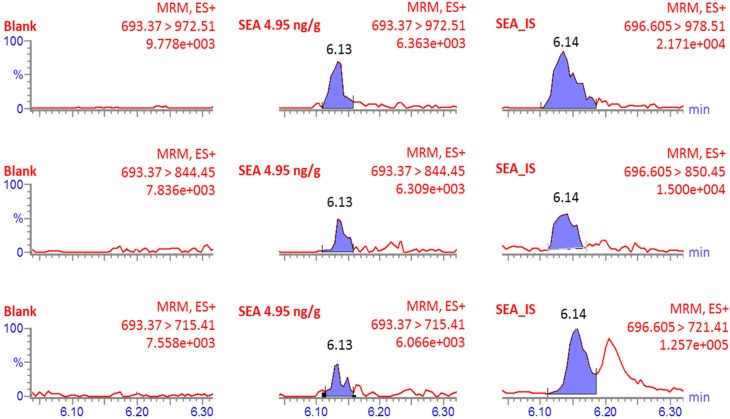
MRM-MS spectra from quantitative analysis of SEA in milk samples. The concentration of SEA is the nominal concentration. IS is the abbreviation for internal standard. No smoothing function was applied to the shown signals. The results from the analysis are presented in Table 6.

In another experiment, the performance of the method was investigated by comparative analysis of milk samples spiked with SEA at concentrations designed for analysis with MS. The samples were kindly provided by the EU reference laboratory (EU-RL) for coagulase positive staphylococci (CPS), (France). The samples were analyzed both at the National Food Agency (NFA, Sweden), using the present method and at the EU-RL using the EU approved immuno-affinity based method, ELISA [16]. The values obtained from the analyses of milk samples did not differ significantly between the two methods (Table 6) confirming the usefulness of the MS-based method in quantitative analysis of staphylococcal enterotoxins in food samples. The two additional test samples, on the other hand, consisting of the matrices for which matrix-matched standards were not used in the MS analysis, did not correlate very well. This indicates the importance of the use of matrix-matched calibration to compensate for signal differences mainly caused by matrix effects in the analysis of complex samples with ESI-MS. No SEA was detected in the corresponding blank samples, Figure 2. The performance of the method is also presented in an overview in Table 7. With this table, it is possible to compare several steps in the already existing LC-MS based methods, regarding matrix complexity, sample preparation comprehensiveness and the choice of standard for the identification confidence, in relation to the quantitative performance of the method. Except for the ideal, but comprehensive and high-cost associated work from Dupuis *et al.* [22], where full-length isotope labeled standards were used in combination with immunoaffinity based toxin extraction, the presented method offers detection and quantification of enterotoxins at low ppb level using relatively straight forward approach and at reasonable costs. This makes the new method more advantageous for frequent use in direct analysis of enterotoxins in foods in comparison to the previously published methods.

**Table 6:** The results obtained for quantitative analysis of SEA in milk using UPLC-ESI-MS/MS method at National Food Agency (NFA, Sweden) and quantitative ELISA at EU-RL for Coagulase Positive Staphylococci (CPS), (ANSES, France). The corresponding blank sample (shown in Figure 2) gave negative result and was not included in the table.

Matrix	Nominal Concentration (ng/g)	UPLC-ESI-MS/MS (NFA) (ng/g)	ELISA (ANSES) (ng/g)
Milk	2.47	2.98	1.80
Milk	4.95	3.55	3.77
Cream dessert	9.89	2.30	9.03
Ready-to-eat-food	0.22	2.68	0.14

**Table 7:** An overview comparing the existing LC-MS based methods with the method presented in this study.

Author of the Method	Matrix	Toxin	Extraction	Detection	Standards	Analyte	LOD	LOQ
Kientz *et al.*, (1997) [35]	Water with sodium phosphate	SEB	Dialysis, digestion	QqQ	N/A	Proteotypic peptides	100 ppb	N/A
Nedelkov *et al.*, (2003) [36]	Mushroom	SEB	Centrifugation, spiking of supernatant, Immunocapture (on sensor chip)	MALDI-TOF	N/A	Whole protein	1 ppb (in extract)	N/A
Callahan *et al.*, (2006) [21]	Apple juice	SEB	UF (MWCO 5 and 10 kDa), digestion	QTOF	Surrogate internal standard.	Proteotypic peptides	60 ppb	100 ppb
				QqQ				
Dupuis *et al.*, (2008) [22]	Cheese, Coco- pearls	13 SEs	Precipitation, Dialysis, immunocapture, SDS-PAGE, in-gel digestion	QTOF	PSAQ (full-length isotope labeled SEs)	Proteotypic peptides	1.5 ppb	1.5 ppb
Sospedra *et al.*, (2011) [23]	Milk	SEA	SDS-PAGE, Digestion	MALDI-TOF	Peptide calibration standards	Proteotypic peptides	N/A	N/A
Bao *et al.*, (2011) [24]	Raw chicken meat	SEB	Protein precipitation, digestion, UF (MWCO 10 kDa)	QIT	Acetic anhydrid label surrogate standards	Proteotypic peptides	6 ppb	6 ppb
Sospedra *et al.*, (2012) [25]	Milk, Apple juice, Orange juice	SEA, SEB	Precipitation	QqQ	Standard curve (external calibration)	Whole protein	25 ppb	50 ppb
Present method	Milk, Shrimps	SEA, SEB	Precipitation, UF (MWCO 100 and 3 kDa), digestion	QqQ	Synthetic ^13^C-labeled proteotypic peptides as internal standards	Proteotypic peptides	2.5 ppb	Milk: 2.5 ppb
								Shrimp: 5 ppb

QqQ, triple quadrupole; MALDI-TOF, matrix assisted laser desorption ionization time-of-flight; UF, ultra filtration; MWCO, molecular weight cut-off; QTOF, quadrupole time-of-flight; SDS-PAGE, sodium dodecyl sulfate polyacrylamide gel electrophoresis; PSAQ, protein standard absolute quantification; QIT, quadrupole ion trap. LOD in Kientz *et al.* [35] and Nedelkov *et al.* [36] were calculated from ng/mL.

## 3. Experimental Section

### 3.1. Reagents

Staphylococcal enterotoxins A and B, Iodoacetamide (IAA, BioUltra), Dithiothreitol (DTT, BioXtra), Urea (BioReagent) and Leucine Enkephalin (Leu-Enk) were purchased from Sigma-Aldrich (Stockholm, Sweden). The synthetic ^13^C_6_-labeled internal standard peptides NVTVQELD(L^13^C_6_)QAR (ISA^13^C_6_) and VTAQELDY(L^13^C_6_)TR (ISB^13^C_6_) were custom-made in Bachem (Bubendorf, Switzerland). Ammonium bicarbonate (NH_4_HCO_3_), pa, was obtained from VWR international (Stockholm, Sweden). Acetonitrile (ACN) of LC-MS grade was purchased from Fischer Scientific (Loughborough, Leicester, UK), and all other chemicals were of pro-analysis grade and obtained from Merck (Darmstadt, Germany). Water was purified with Milli-Q purification system (Millipore, Solna, Sweden). Stock solutions of all the reagents were prepared by dissolving the solid mater in Milli-Q water. The enterotoxins A and B, internal standards and Leu-Enk were prepared as 1 or 0.5 mg/mL stock solutions. All the solutions were stored at −80 °C until analysis when they were further diluted to prepare working solutions: enterotoxins A and B standards at a concentration of 10 μg/mL (10 ng/μL), ISA^13^C_6_ and ISB^13^C_6_ as 1 μg/mL solutions, respectively, and Leu-Enk at a concentration of 10 μg/mL, in water. Trypsin (Gold, mass spectrometry grade) was purchased from Promega Biotech (Stockholm, Sweden) and prepared according to instructions of the manufacturer in a concentration of 1 μg/μL by dissolving 100 μg of trypsin powder in 100 μL of 50 mM acetic acid (HAc). The solution was aliquoted and stored at −80 °C until usage. The tryptic digestion reagents solutions, DTT 45 mM and IAA 100 mM were prepared in water as well as 12 M urea in 0.4 M NH_4_HCO_3_.

### 3.2. Materials

A laboratory blender Stomacher 400 (VWR), with paddles applied pressure and double bags, was used for homogenization of solid samples. Centrifugal filters of the type Centricon^®^ Plus-70 (Merck Chemicals and Life Science, Stockholm, Sweden) with two different membrane nominal molecular weight limits (NMWL), 3 and 100 kDa were used, with the compatibility for processing biological aqueous solutions and the volumes of 15–70 mL. The filters were washed prior to use with water to remove glycerin in the filter membrane and used according to instructions and recommendations of the manufacturer. Solid phase extraction (SPE), was performed using Isolute_18_ (EC) (1 mL, 50 mg capacity, Biotage, Uppsala, Sweden) SPE columns to clean the tryptic peptides. A SpeedVac concentrator, Savant SPD 2010 system (Thermo Fisher Scientific, Asheville, NC, USA) was used for drying SPE eluates. The dry sample was redissolved in a mixture of mobile phases, 80% of the mobile phase A and 20% of mobile phase B, (A 80%/B 20%) prior to the UPLC-ESI-MS/MS analysis.

### 3.3. Sample Preparation

#### 3.3.1. Extraction and Concentration of Enterotoxins

Cow-milk (3% fat) and shrimp (without shell) were used as model samples. Initial part of the sample preparation procedure was performed according to the official method (approved by European Commission) [32], which in the second part was modified by substituting the dialysis step with centrifuge driven ultrafiltration. The sample preparation procedure used in this study was as follows: 25 g of milk or shrimp sample was used. For preparing calibration curves, samples were spiked with SEA and SEB at five concentration levels: 2.5, 5, 10, 15 and 30 ng/g. To the samples of shrimp, where the toxins were spiked into the solid matrix, 40 mL of 40 °C Milli-Q water was added following the homogenization using a stomacher blender for 30 s. Milk samples were further treated without addition of water. The samples were shaken for 45 min where after the pH in the sample was adjusted to 3.5 ± 5 with HCl following a centrifugation step at 3200 RCF and 4 °C for 15 min. The supernatant was transferred to a new centrifuge flask and the pH was neutralized to 7.4–7.6 using NaOH following a second centrifugation step as described above. Acetonitrile was added to the supernatant (corresponding to ~0.5% *v*/*v* of the extract) to break protein–protein interactions and thereby prevent clogging of the filter. The supernatant was mixed on vortex for 20 s and further processed through a 100 kDa NMWL centrifuge filter at 3500 RCF and 25 °C. The filtrate was then transferred to a 3 kDa NMWL centrifuge filter and processed in the same way as in the previous filtration step above. The retentate, corresponding to a volume of approximately 0.5 mL, was recovered and spiked with internal standard (ISA^13^C_6_ and ISB^13^C_6_) at a concentration of 15 ng/g, respectively.

#### 3.3.2. Enzymatic Digestion and Cleaning

**Figure 3:**
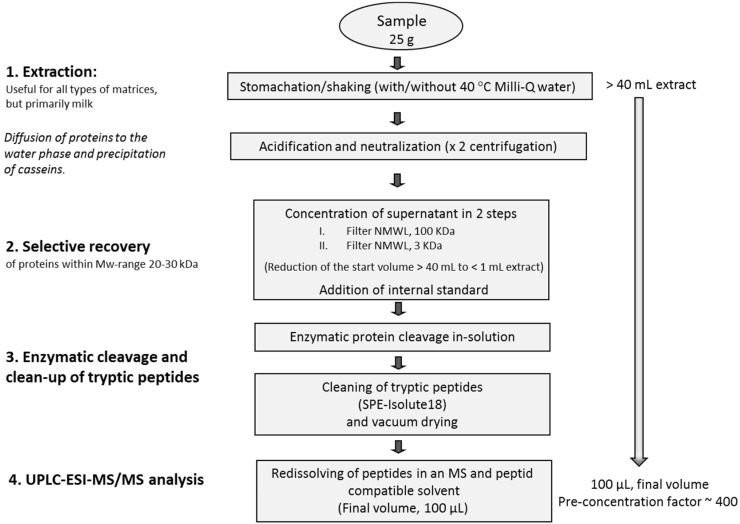
Summarizing flow chart of the sample preparation procedure and analysis of SEA and SEB.

The sample was mixed with urea in 0.4 M NH_4_HCO_3_ so that the final urea concentration in the sample was 6 M, where after 10 μL of 45 mM DTT was added. The sample was kept at 50 °C for 15 min. After cooling to room temperature, 10 μL of 100 mM IAA was added and the sample was incubated for 15 min in darkness. Finally, trypsin was added to give a trypsin: protein ratio of approximately 2% (*w/w*) and the sample was incubated upon light shaking at 60 °C for 16 h. The digested sample was cooled to room temperature, acidified (to pH ≤ 4) with acetic acid (HAc) and cleaned with Isolute_18_ SPE columns. The SPE column was first washed with 5 × 1 mL 100% ACN and equilibrated with 5 × 1 mL 1% HAc. The tryptic peptides were adsorbed to the media using 5 repeated cycles of sample loading. The column was washed using 5 × 1 mL of 1% HAc and finally the peptides were eluted in 250 μL 50% ACN, 1% HAc. After the cleaning, the eluate was vacuum centrifuged to dryness and redissolved in a mixture of mobile phases, 80% of the mobile phase A and 20% of mobile phase B, (A 80%/B 20%) prior to the UPLC-ESI-MS/MS analysis. Additionally, standard proteins of SEA and SEB were mixed in Milli-Q water, after which the digestion, cleaning, drying and redissolving proceeded as described above to prepare a standard peptide mixture at a concentration of 10 ng/μL for each of the toxins after redissolving. Serial dilutions of this standard peptide mixture of SEA and SEB digest peptides were used for preparation of standard curves (2.5–30 ng/g) in mobile phase mixture at proportions described in Section 3.2 (these solutions of standard digest in mobile phase mixture are referred to as “standard digest in water” in the rest of the article). When a complete standard curve in water was not needed, at least one of the toxin digest solutions was continuously used to check the maintaining of the optimal experimental conditions for the analysis of enterotoxins in food samples. The experimental overview of the method set-up is shown by a flow chart in Figure 3.

### 3.4. UPLC-ESI-MS/MS

Analysis was performed using an Acquity UPLC BEH C18 1.7 μm, 2.1 × 100 mm column with an Acquity UPLC BEH 300, C18, 1.7 μm, 2.1 × 5 mm guard column and Waters UPLC I-Class (Waters, Milford, MA, USA) with Waters Xevo TQ-S mass spectrometer system (Waters, Milford, MA, USA) operating in ESI+ mode. The ionization parameters were set to: capillary voltage 3.2 kV, desolvation temperature 350 °C, desolvation gas flow rate 600 L/h, source temperature of 150 °C, and the cone gas flow rate 150 L/h. The column temperature was maintained at 45 °C and the injection volume was 5 μL. The analysis was performed in multiple-reaction-monitoring mode and argon was used as the collision gas. Mobile phase gradient consisted of 0.1% formic acid in water (A) and 0.1% formic acid in acetonitrile (B). The flow rate was set to 0.5 mL/min. The gradient started at 1% B and linearly increased to 50% B over 15 min, then increased to 100% B over 0.5 min and kept at 100% B for 1.5 min where after it was reduced to 1% B over 0.5 min, following an equilibration period of 1.5 min. SIM studies were primarily performed on all standard digest peptide masses higher than 500 Da, predicted by *in silico* digest using UniProt database [37]. The dwell times were set to 0.03 s, and the span was 1 amu. MRM was further conducted to optimize the fragmentations and to confirm the retention times for the selected peptides. For each precursor peptide ion 3–5 fragment ions were monitored. For the identification in matrices the most abundant toxin specific peptides of SEA and SEB, selected as precursor ions in MS1 and their corresponding product ions in MS2, are presented in Table 1 and Table 2 with the settings of the MS and MS/MS analysis summarized in details. The bold sequences indicate the peptides with the highest abundance used for quantification in MS2. For each of these two toxin specific peptides a synthetic ^13^C_6_-labeled internal standard peptide was used. For quantitative analysis Targetlynx v 4.1 software (Waters, Milford, MA, USA, 2011) was applied. SEA and SEB spiked milk and shrimp matrices (2.5, 5, 10, 15 and 30 ng/g) prepared according to the procedure described in Section 3.3, all containing ^13^C_6_-internal standards at 15 ng/g, were injected to obtain calibration curves. These were constructed by plotting peak area ratios of the enterotoxin to internal standard against concentration ratios of the analyte to the internal standard using linear regression. For the confirmation of the analyte identification, an amino acid sequence coverage of 10% for the protein was aimed [38] corresponding to approximately 25 amino acids in SEA and SEB, respectively. In the present study, the minimum sequence coverage of 10% was achieved by monitoring three peptide precursor ions with the corresponding product ions for each of the toxins (peptides indicated by bold and underlined sequences in Table 1 and Table 2). For the confirmatory peptide precursor ions (the underlined sequences) at least one product ion was required. To ensure the absence of co-eluting substances the ion ratios in the spiked samples were compared to those of the standard digest in water. For the quantification peptide at least two product ions were required and for the other two peptides (the underlined sequences) at least one product ion in MS2, at the signal-to- noise ratio 3:1 (calculated from the measurement of the peak-to-peak noise around the retention time of the analyte). A Leu-Enk solution of 1 μg/mL in water and in the matrix was used as a system performance sample (SPS) to screen for the daily condition of the UPLC-ESI-MS/MS system. This was done by utilizing Leu-Enk as a retention time and area shifting marker. In addition, at least one solution of enterotoxin digest peptides in water (e.g., corresponding to 15 ng/g) was used to confirm the system stability. These three samples were injected according to the bracketing principle during the analysis of a whole sample batch, which together with the ^13^C_6_-internal standards in the samples provided a reliable base for the determination of the retention time for target peptides.

## 4. Conclusions

LC combined with tandem MS using multiple reaction monitoring (MRM) is regarded to be a highly specific analytical technique. This especially holds true for the determination of analytes with a comparatively high molecular weight range, like proteins and peptides (Q1 masses of tryptic peptides usually range between *m*/*z* 500 and *m*/*z* 2300). In the present method development, a proteomics-based bottom-up approach was used to identify and quantify SEA and SEB in the food matrices milk and shrimp. Sample extract sizes of ≥40 mL were volume reduced approximately 400 times allowing for 2.5 and 5 ng/g levels of SEA and SEB in milk and shrimp, respectively, to be detected and quantified by MS/MS on a triple-quadrupole mass spectrometer. Thus, the method can be applied for direct identification of SEA and SEB in samples that induce illness in humans. The presented detection level has been achieved thanks to a substantial sample complexity reduction where many of the interfering components are removed by selective fractionation using centrifuge driven ultrafiltration and solid phase extraction. The low detection level was achieved despite a low analyte recovery (<8%) and pronounced competitive matrix suppression in the electrospray ionization. This, consequently, leaves possibilities open for improvements in sample preparation and the recovery. This work has also shown a successful use of ^13^C-labeled synthetic internal standard peptides together with the corresponding target peptides in quantitative determination of SEA and SEB toxins, an approach applicable for quantification of multiple enterotoxins for relatively affordable costs. Although the present method was developed and validated for analysis of SEA and SEB in two specific high-protein-content food matrices, it is likely to be applicable in principle for the analysis of other staphylococcal enterotoxins and in a wide range of food matrices.
